# Identification of a new prognostic score for patients with high-grade metastatic GEP-NEN treated with palliative chemotherapy

**DOI:** 10.1007/s00432-022-04314-5

**Published:** 2022-09-08

**Authors:** Vivian Rosery, Stephan Mika, Kurt Werner Schmid, Henning Reis, Martin Stuschke, Jürgen Treckmann, Peter Markus, Brigitte Schumacher, David Albers, Bastian Mende, Harald Lahner, Marcel Wiesweg, Martin Schuler, Jens T. Siveke, Stefan Kasper

**Affiliations:** 1 Department of Medical Oncology, West German Cancer Center, University Hospital Essen (AöR), Essen, Germany; 2 Bridge Institute of Experimental Tumor Therapy, West German Cancer Center, University Hospital Essen (AöR), Essen, Germany; 3 Institute of Pathology, West German Cancer Center, University Hospital Essen (AöR), Essen, Germany; 4 Department of Radiotherapy, West German Cancer Center, University Hospital Essen (AöR), Essen, Germany; 5 General, Visceral and Transplantation Surgery, University Hospital Essen (AöR), Essen, Germany; 6 Department of General Surgery and Traumatology, Elisabeth Hospital Essen, Essen, Germany; 7 Department of Gastroenterology, Elisabeth Hospital Essen, Essen, Germany; 8 Central Pharmacy, University Hospital Essen (AöR), Essen, Germany; 9 Department of Endocrinology and Metabolism, University Hospital Essen (AöR), Essen, Germany; 10 Division of Solid Tumor Translational Oncology, German Cancer Consortium (DKTK), Partner site University Hospital Essen, and German Cancer Research Center (DKFZ), Heidelberg, Germany; 11 German Cancer Consortium (DKTK), Partner Site University Hospital Essen (AöR), Essen, Germany; 12 Present Address: Institute of Pathology, University Hospital Frankfurt, Frankfurt, Germany

**Keywords:** Gastroenteropancreatic neuroendocrine neoplasm, Systemic inflammatory response marker, Prognostic factor, Survival analysis

## Abstract

**Purpose:**

High-grade gastroenteropancreatic neuroendocrine neoplasms (GEP-NEN G3) are rare and heterogeneous malignancies with poor prognosis. Aim of this study was to develop prognosticators identifying those patients that derive the most benefit from currently available systemic therapies.

**Methods:**

This retrospective analysis included 78 patients with metastatic GEP-NEN G3. For patients with imaging data available (*n* = 52), the overall response rate (ORR) and disease control rate (DCR) were evaluated according to the Response Evaluation Criteria in Solid Tumors 1.1 (RECIST 1.1). A Cox proportional hazard model was used to analyze the prognostic value of selected clinical and blood-based biomarkers. The impact of palliative chemotherapy regimens on time-to-treatment-failure (TTF) and overall survival (OS) was assessed.

**Results:**

Median OS of the study cohort was 9.0 months (95% CI 7.0–11.1). The majority of patients received first-line treatment with platinum plus etoposide (83.3%). The ORR and DCR of the RECIST-evaluable subgroup were 34.6% and 76.9%. Median TTF upon first-line treatment was 4.9 months (95% CI 3.4–6.4). Multivariate analysis identified the Eastern Cooperative Oncology Group performance status (ECOG PS), lactate dehydrogenase (LDH) and absolute lymphocyte count as independent prognostic factors. A prognostic score based on these parameters discriminated patients with favorable and unfavorable outcomes.

**Conclusion:**

Outcomes of patients with GEP-NEN G3 are still limited. A new prognostic score identifying those patients benefitting from current platinum/etoposide-based chemotherapy protocols may help as stratification factor in future trial design.

**Supplementary Information:**

The online version contains supplementary material available at 10.1007/s00432-022-04314-5.

## Introduction

Gastroenteropancreatic neuroendocrine neoplasms (GEP-NEN) are a heterogeneous group of malignancies emerging from the diffuse neuroendocrine cell system in the gastrointestinal tract (Ilett et al. 2015; Garcia-Carbonero et al. 2016). The incidence of GEP-NEN has increased over the past decades (Dasari et al. 2017). According to the updated WHO classification of 2019, GEP-NEN are classified based on morphology and proliferation rate into well-differentiated tumors (NET G1–G2: Ki-67 ≤ 20%; NET G3: Ki-67 > 20%) and poorly differentiated, clinically highly aggressive neuroendocrine carcinomas (NEC, always G3: Ki-67 > 20%) (Lloyd 2017; Nagtegaal et al. 2019). NET G3 and NEC have a different clinical behavior concerning prognosis and treatment response. Patients with GEP-NET G3 have lower response rates with platinum-based chemotherapy, but in general have a better prognosis. However, the GEP-NEN G3 group includes both NEC and NET G3 (Sorbye et al. 2019).

GEP-NEN G3 is an “orphan-disease” and data concerning the best treatment strategy particularly in the palliative metastatic setting is limited. Treatment is challenging due to the aggressive tumor biology, early metastasis and primary resistance to multiple cytotoxic drugs (Garcia-Carbonero et al. 2016). Currently, first-line chemotherapy with a platinum compound in combination with etoposide is recommended at least for patients with NEC and fast growing (Ki-67 ≥ 55%) NET G3 (Shah et al. 2021). This treatment recommendation is based on the treatment paradigm for patients with small-cell lung cancer (SCLC) as both diseases have comparable clinical and biological behavior (Sorbye et al. 2014). For GEP-NEN G3, oxaliplatin plus fluoropyrimidines, i.e., FOLFOX, is a less toxic alternative option. Unfortunately, response rates to first-line therapies are only about 30 to 50% and there is no established second-line option (Sorbye et al. 2013). Consequently, survival of patients with high-grade GEP-NEN did not improve over the last decades and new therapeutic strategies are urgently needed (Ilett et al. 2015).

Immunotherapeutic approaches, such as the immune checkpoint blockade (ICB), have changed the therapeutic landscape of many cancer entities (Wei et al. 2018). Treatment efficacy of PD-1/PD-L1 inhibition in early clinical trials with GEP-NEN G3 has been disappointing and no predictive biomarkers have been established so far (Giannetta et al. 2021). However, there is still only limited evidence of efficacy of ICB in NEN (Weber and Fottner 2018; Bongiovanni et al. 2021).

Chronic inflammation plays a critical role in the development and progression of cancer (Fridman et al. 2012). Besides environmental factors, malignant cells themselves promote inflammation by recruitment and activation of immune cells in the tumor microenvironment (Colotta et al. 2009; Grivennikov et al. 2010; Todoric et al. 2016). Tumor growth induces a systemic inflammatory response, which can be reflected by elevated circulating white blood cells and acute phase proteins in the peripheral blood. These systemic inflammatory response (SIR) markers, such as the absolute lymphocyte and neutrophil count, C-reactive protein (CRP), neutrophil–lymphocyte ratio (NLR), lymphocyte–monocyte ratio (LMR), platelet–lymphocyte ratio (PLR), are routinely assessed in clinical settings prior to treatment and are associated with patients’ outcome in many malignancies, independently of tumor stage (Roxburgh and McMillan 2014; Dolan et al. 2017).

Against this background, we analyzed a cohort of GEP-NEN G3 patients to explore whether specific clinical or laboratory-based parameters may associate with outcome and treatment response in this rare cancer entity.

## Patients and methods

### Study design and assessment

In this retrospective study, we evaluated the outcome of patients with histologically confirmed, metastatic, high-grade gastro-entero-pancreatic neuroendocrine neoplasms (GEP-NEN G3, Ki-67 > 20%, thus including both NET G3 and NEC) treated with palliative chemotherapy at the West German Cancer Center, University Hospital Essen, between January 2010 and April 2019. Data were extracted from the electronic health record (EHR). All data were anonymized for further analysis. Patients were evaluable, if a predefined set of pretreatment laboratory parameters was available and if they had received at least one dose of palliative chemotherapy. This retrospective study was approved by the local ethics committee of the Medical Faculty of the University Duisburg-Essen (No. 17-7472-BO).

The American Joint Committee on Cancer (AJCC)/International Union Against Cancer (UICC) TNM classification (7th Edition) was used for staging. Only patients with metastatic disease, UICC stage IV, were included. Clinical staging was based on EHR data or if available on computed tomography (CT) or magnetic resonance imaging (MRI). Overall response rate (ORR) and disease control rate (DCR) were evaluated according to the Response Evaluation Criteria in Solid Tumors 1.1 (RECIST 1.1) (Therasse et al. 2000; Eisenhauer et al. 2009). ORR was defined as complete or partial remission on the applied chemotherapy regiment and DCR was defined as complete or partial remission or disease stabilization. Response evaluation by RECIST 1.1 was performed as best overall response rate (BORR) if at least one baseline CT or MRI (maximum of 8 weeks prior to start of palliative chemotherapy) and one follow-up imaging upon chemotherapy (minimum of 6 weeks after start of palliative treatment) was available. Under palliative chemotherapy, most patients underwent imaging studies in 8–12 week intervals. Time-to-treatment-failure (TTF) was defined as time from start of palliative chemotherapy to date of radiological or clinical progression, change of treatment regimen or death. Overall survival (OS) was defined as time from start of palliative chemotherapy to death. If time of death was unknown, patients were censored at the time of last follow-up.

### Pretreatment clinical and serum parameters

Clinical data and peripheral blood parameters within a maximum of 2 weeks before the start of palliative chemotherapy were extracted from the EHR. Based on previously published studies, we focused on five potentially prognostic clinical parameters: gender, age, ECOG performance status (PS), Ki-67 fraction and lactate dehydrogenase activity (LDH). Six systemic inflammatory response (SIR) markers were assessed: absolute lymphocyte and neutrophil counts, C-reactive protein (CRP), neutrophil–lymphocyte ratio (NLR), lymphocyte–monocyte ratio (LMR), platelet–lymphocyte ratio (PLR). The NLR was defined as the absolute blood neutrophil count divided by the absolute lymphocyte count. The PLR was defined as the absolute platelet count divided by the absolute lymphocyte count. The LMR was defined as the absolute lymphocyte count divided by the absolute monocyte count. The median served as cut-off for age and the assessed SIR markers and patients were grouped into “high” and “low” according to the median (Suppl. Table 1). ECOG was dichotomized between PS 0–1 and ≥ 2. LDH elevation was defined as an increase of at least 1.5 × above upper limit of normal (ULN). Based on the data of the NORDIC NEC study, the Ki-67 cut-off was set at 55% (Sorbye et al. 2013).

### Statistical analysis

Statistical analyses were performed using SPSS Statistics Version 27.0 (IBM, Armonk, NY, USA) and Microsoft Excel Version 16.6 (Microsoft, Richmond, WA, USA). The plot for sequential therapy lines was produced using R 3.6, the tidyverse and the package ggalluvial. The impact of all clinical and blood-based parameters on OS was analyzed using Kaplan–Meier calculations and univariate Cox proportional hazard-analysis. Kaplan–Meier survival analyses were done using the log-rank (Mantel–Cox) test. Univariate and multivariate analyses were performed using a Cox proportional hazard model. Hazard ratios (HR) and 95% confidence intervals (CI) were indicated. Differences in overall response were evaluated using the Fisher’s exact test. Overall, *P* values < 0.05 were considered statistically significant.

## Results

### Patients’ characteristics

We identified 78 evaluable patients with metastatic, highly proliferative GEP-NEN G3 (Ki-67 > 20%) treated at the West German Cancer Center (WTZ) between January 2010 and April 2019. The baseline characteristics of the study cohort are summarized in Table 1.

**Table 1:** Baseline characteristics of the study cohort (*N* = 78)

	%	*N*
Gender		
Female	46.2	36
Male	54.8	42
Median age		
62 years (range: 32–81 years)		
ECOG performance status		
0–1	75.6	59
≥ 2	24.4	19
Histology		
NEN G3	92.3	72
MiNEN	7.7	6
Ki-67		
> 55%	66.7	52
21–55%	33.3	26
Median Ki-67		
77.5% (range: 21–100%)		
Primary location		
Esophagus	7.7	6
Gastric	5.1	4
Small intestine	11.5	9
Pancreaticobiliary	16.7	13
Colorectal	25.6	20
Unknown^a^	33.3	26
Sites of metastasis		
Liver	85.6	66
Lymph nodes	56.4	44
Lung	16.7	13
Peritoneum	15.4	12
Other^b^	28.2	22
Median number of metastatic sites		
2 (range: 1–5)		

*ECOG* Eastern Cooperative Oncology Group, *NEN* neuroendocrine neoplasm, *MiNEN* mixed neuroendocrine non-neuroendocrine neoplasms

^a^Cancer of unknown primary (CUP) with main tumor burden in the abdomen

^b^Bone, brain, pleura, kidney, adrenal gland, pancreas and skin

We included 42 males and 36 females, with a median age of 62 years (range: 32 to 81 years). In total, 59 patients (75.6%) had an ECOG PS of 0–1. The majority (92.3%) had histologically confirmed high-grade GEP-NEN according to the WHO criteria at time of biopsy (Bosman 2010; Lloyd 2017). A group of six (7.7%) mixed neuroendocrine non-neuroendocrine neoplasms (MiNEN) were included. In all 6 MiNEN (7.7%), the non-neuroendocrine component was an adenocarcinoma (mixed adeno-neuro-endocrine carcinoma; MANEC). The primary tumors were located in the colorectum (*N* = 20), pancreas (*N* = 13), esophagus (*N* = 6), stomach (*N* = 4) or in the small intestine (*N* = 9). Patients with unknown primary tumor (“cancer of unknown primary”; CUP) were included if the main tumor burden was localized in the abdomen (*N* = 26). The most prevalent metastatic sites were liver (*N* = 66), lymph nodes (*N* = 44), lung (*N* = 13) and the peritoneum (*N* = 12). Patients had in median two metastatic sites before start of palliative treatment (range: 1–5). Median proliferative activity (Ki-67) was 77.5% (range: 21–100%); 52 patients (66.7%) had a Ki-67 > 55% and 26 patients (33.3%) had a Ki-67 between 21 and 55%.

### Palliative therapy

**Fig. 1:**
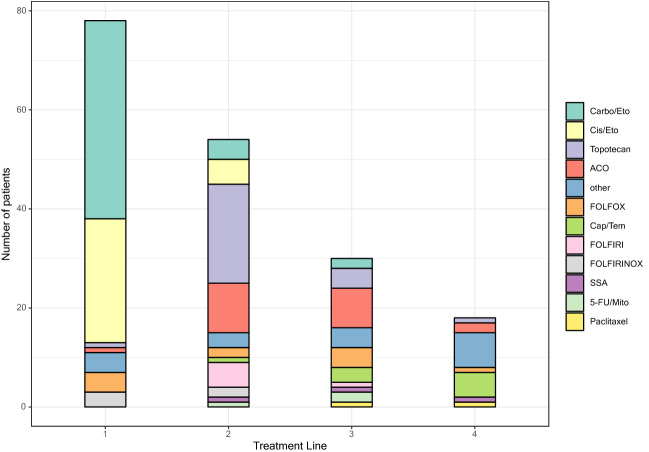
Sequential palliative chemotherapy regimen up to fourth-line treatment. Carbo/Eto carboplatin/etoposide, Cis/Eto cisplatin/etoposide, ACO doxorubicin, cyclophosphamide, vincristine, FOLFIRINOX oxaliplatin, irinotecan, fluorouracil and leucovorin, FOLFOX oxaliplatin, fluorouracil and leucovorin; FOLFIRI irinotecan, fluorouracil and leucovorin, Cap/Tem capecitabine/temozolomide, SSA somatostatin analog, 5-FU/Mito fluorouracil and mitomycin

The majority of patients (*N* = 65, 83.3%) received platinum-based first-line chemotherapy (cisplatin or carboplatin) in combination with etoposide. Alternative first-line therapies included FOLFOX/FOLFIRINOX (*N* = 8, 12.3%) and others. In total, 54 patients (69.2%) received second-line treatment, and 30 patients (38.5%) received further-line therapies (third-line therapy: *N* = 30, 38.5%; ≥ fourth-line therapy: *N* = 18, 23.1%). The most common second-line regimens were topotecan (*N* = 20, 37.0%), and doxorubicin/cyclophosphamide/vincristine (ACO, *N* = 10, 18.5%). ACO (*N* = 8, 26.7%) and FOLFOX/FOLFIRI (*N* = 5, 16.7%) were the most common third-line treatments. Variable protocols were selected for further-line therapy (Fig. 1, Suppl. Table 2).

### Treatment outcome

**Fig. 2:**
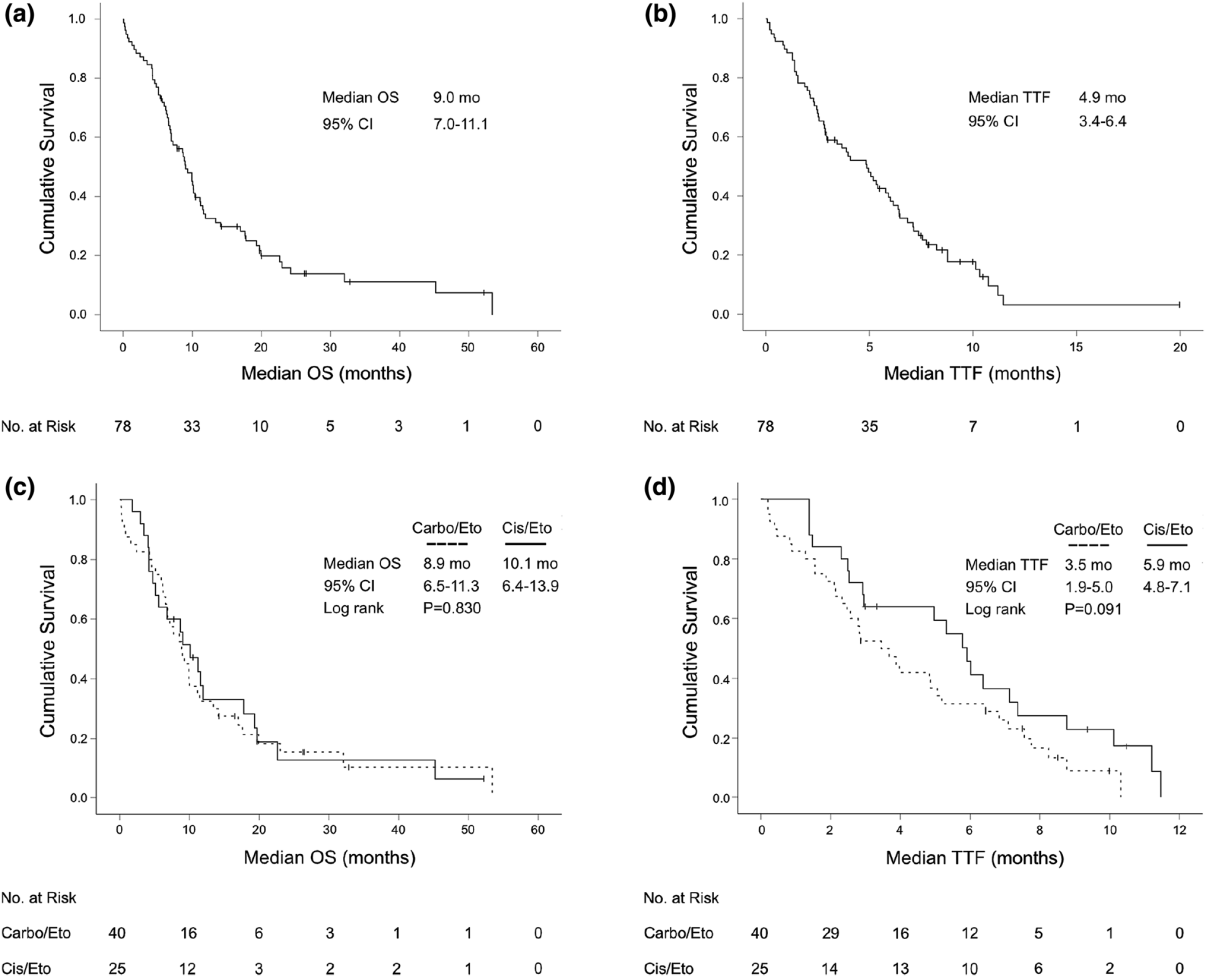
Kaplan–Meier plot for overall survival (OS) and time-to-treatment-failure (TTF) from start of palliative fist-line therapy for the whole study cohort (**a**, **b**) and for patients receiving carboplatin/etoposide or cisplatin/etoposide as first-line treatment (**c**, **d**)

The median OS from start of palliative treatment was 9.0 months (95% CI 7.0–11.1) for the entire study cohort. The median follow-up time was 10.7 months (range: 0.03–54 months). A total of 65 patients (83.3%) had died during follow-up. 2 patients (2.6%) were lost to follow-up. The median TTF upon first-line treatment was 4.9 months (95% CI 3.4–6.4). There was no significant difference in median OS (*P* = 0.830) and TTF (*P* = 0.091) between carboplatin/etoposide and cisplatin/etoposide. Further, a statistical superiority of TTF under platinum/etoposide-based first-line therapy over alternative first-line protocols could not be established (*P* = 0.191). The median TTF of second-, third- and further-line therapies were 1.7, 2.1 and 1.8 months, respectively (Fig. 2, Table 2).

**Table 2:** TTF upon sequential palliative chemotherapy and median OS for the study cohort (N = 78)

Median TTF	Months (CI 95%)	*P* value
1st line	4.9 (3.4–6.4)	
Platinum/Etoposide	4.8 (3.1–6.5)	0.091 (Carbo/Eto vs Cis/Eto)
Carboplatin/Etoposide	3.5 (1.9–5.0)	
Cisplatin/Etoposide	5.9 (4.8–7.1)	
Other	6.1 (2.7–9.6)	0.191 (Platinum/Eto vs Others)
2nd line	1.7 (1.2–2.3)	
3rd line	2.1 (1.6–2.5)	
4th line	1.8 (1.4–2.2)	

Median OS	Months (CI 95%)
Since diagnosis	11.0 (9.3–12.7)
Since palliative care	9.0 (7.0–11.1)
**Follow-up time**	10.7 months (range 0.03–54 months)
**Number of cases lost to follow-up**	*N* = 2 (2.6%)
**Death during follow-up**	*N* = 65 (83.3%)

*TTF* time-to-treatment-failure, *Carbo* carboplatin, *Cis* cisplatin, *Eto* etoposide, *OS* overall survival

From the entire study population, 52 patients (66.7%) were evaluable for response analysis according to RECIST 1.1, including 47 patients treated with platinum/etoposide and 5 patients receiving alternative protocols. The ORR of first-line therapy was 34.6% and the DCR 76.9% at time of best response. As expected, ORR of second- and further-line chemotherapies was lower (Suppl. Table 3a). Patients with a Ki-67 > 55% (*N* = 29, ORR 48.3%) had a significantly higher ORR to platinum/etoposide first-line therapy as compared to patients with a lower Ki-67 (*N* = 18, ORR 5.6%) (*P* = 0.003). DCR for both groups were 82.8% and 61.1%, respectively (Suppl. Table 3b).

**Fig. 3:**
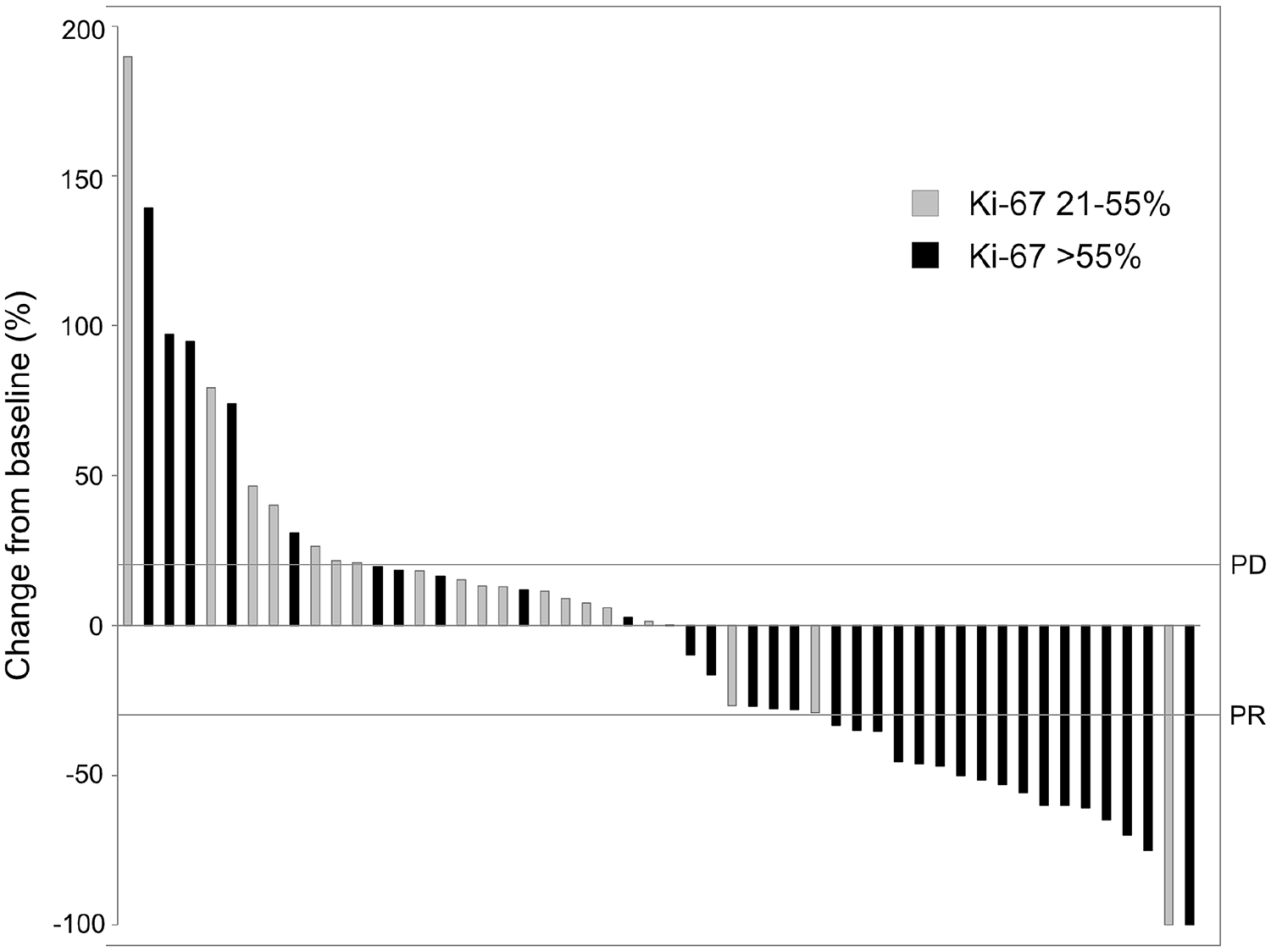
Waterfall plot for target lesion diameters at time of best response to palliative first-line chemotherapy assessed by RECIST 1.1 (*N* = 52)

**Fig. 4:**
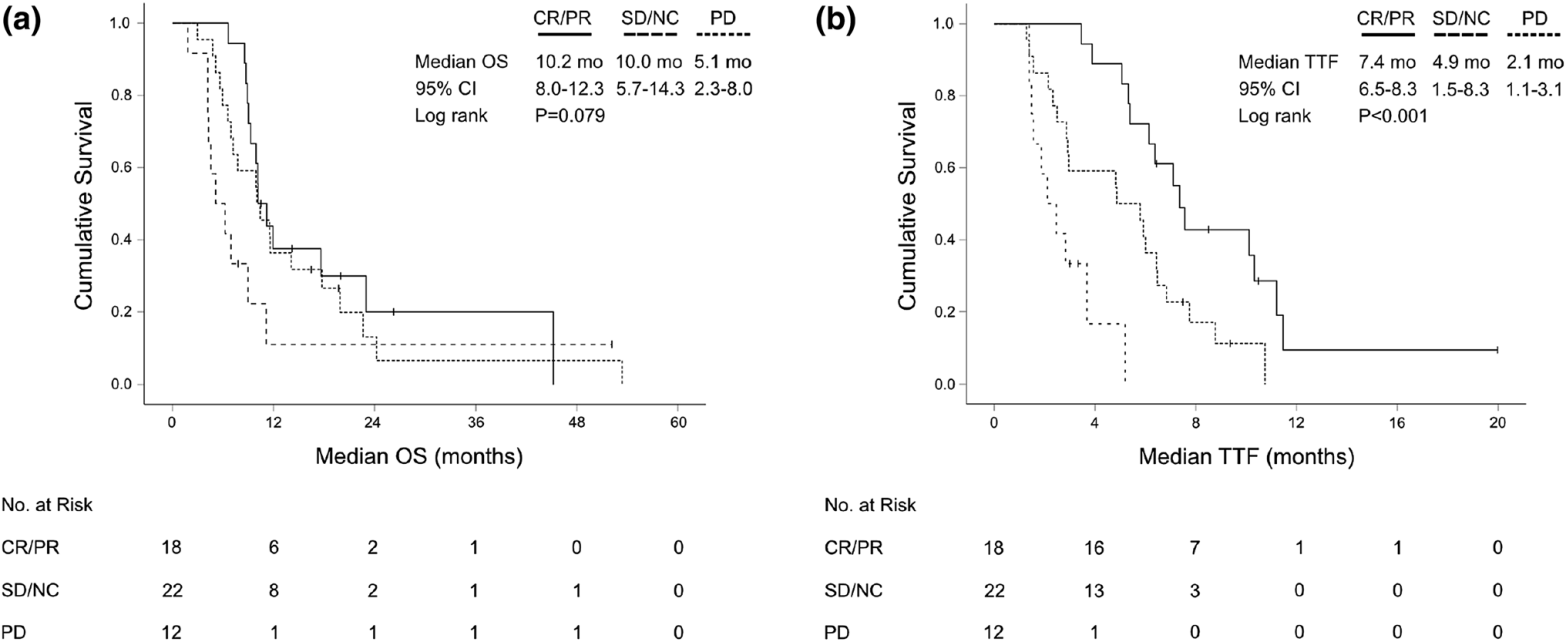
Kaplan–Meier plot for **a** overall survival (OS) since start of first-line palliative chemotherapy and **b** time-to-treatment-failure (TTF) upon start of first-line treatment for patients achieving a complete or partial response (CR/PR), stable disease (SD/NC) or progressive disease (PD) according to RECIST 1.1

In total, tumor shrinkage was observed in 25 patients (48.1%) with first-line palliative treatment. The median tumor shrinkage at time of best response was -46.9% (range: − 9.8 to − 100) (Fig. 3). The median TTF of patients who responded to first-line therapy (CR/PR) was significantly longer compared to those only achieving stable disease (SD/NC) (7.4 months, 95% CI 6.5–8.3 vs 4.9 months, 95% CI 1.5–8.3; *P* < 0.001). However, this did not translate into a significant OS advantage (*P* = 0.443). Patients who achieved CR/PR still had superior OS compared to those with primary progression (*P* = 0.024). As expected, patients with progressive disease upon first-line therapy had significantly inferior median OS compared to treatment responders achieving CR/PR or SD (5.1 months, 95% CI 2.3–8.0 vs 10.2 months, 95% CI 8.3–12.0; *P* = 0.032) (Fig. 4).

### Prognostic impact of clinical parameters and serum markers of inflammation (SIR)

**Fig. 5:**
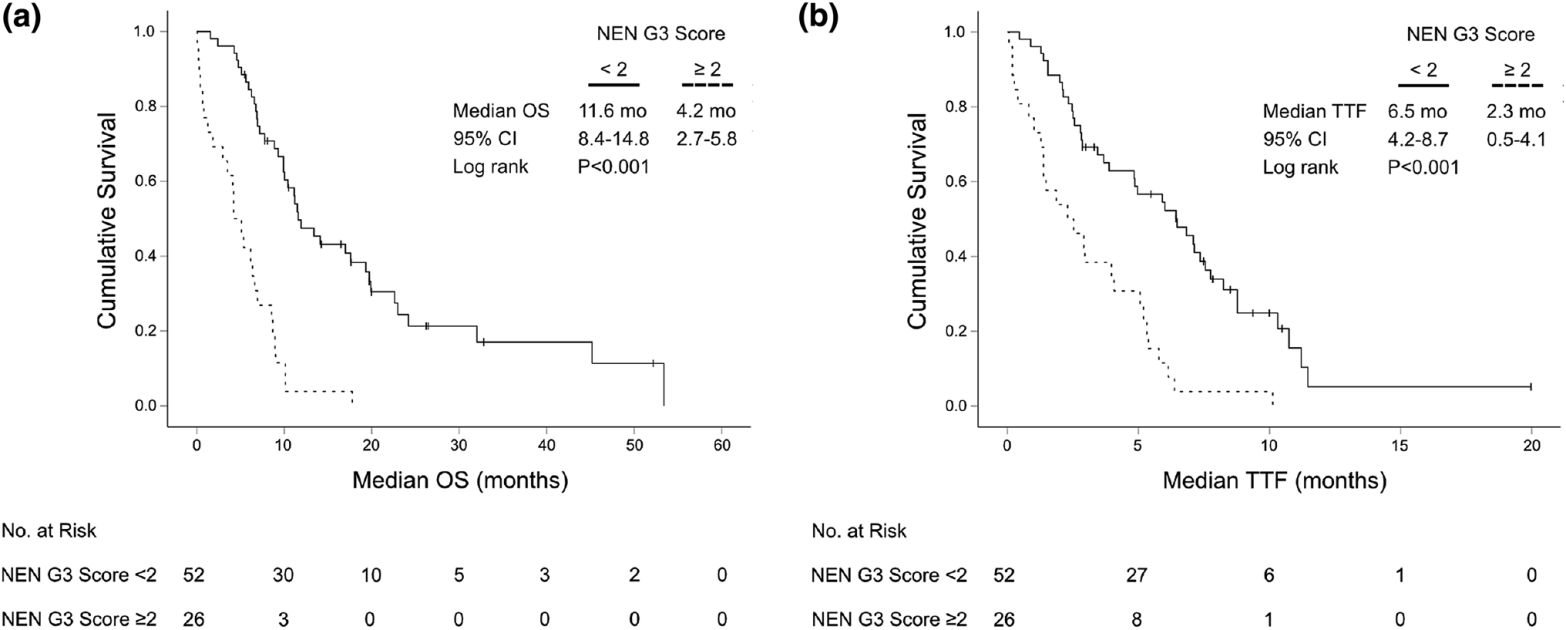
Kaplan–Meier plot for **a** median overall survival (OS) and **b** time-to-treatment-failure (TTF) for patients with a high (≥ 2 prognostic factors) and low (< 2 prognostic factors) “NEN G3 Score” (*N* = 78)

At univariate analysis ECOG, Ki-67 index, LDH and all SIR markers correlated with OS. In particular, a low absolute lymphocyte count was significantly associated with reduced median OS (6.9 months, 95% CI 6.0–7.8 vs 11.9 months 95% CI 6.8–17.0; *P* = 0.001) (Suppl. Figure 1). In multivariate analysis, ECOG ≥ 2, LDH ≥ 1.5 ULN and a low absolute lymphocyte count (< median) emerged as independent adverse prognostic markers (Suppl. Figure 2). A new “NEN G3 Score” was developed based on these independent prognostic parameters, with one point assigned for every parameter that was met (0–3 points in total). Patients with a NEN-G3 Score ≥ 2 had a significantly reduced median OS (4.2 months, 95% CI 2.7–5.8) as compared to patients with a NEN G3 Score < 2 (11.6 months, 95% CI 8.4–14.8) (HR 4.9, 95% CI 2.8–8.6, *P* < 0.001). Moreover, median TTF was significantly reduced for patients with NEN-G3 Score ≥ 2 (2.3 months, 95% CI 0.5–4.1 vs 6.5 months, 95% CI 4.2–8.7) (HR 3.2, 95% CI 1.9–5.4, *P* < 0.001) (Fig. 5).

**Fig. 6:**
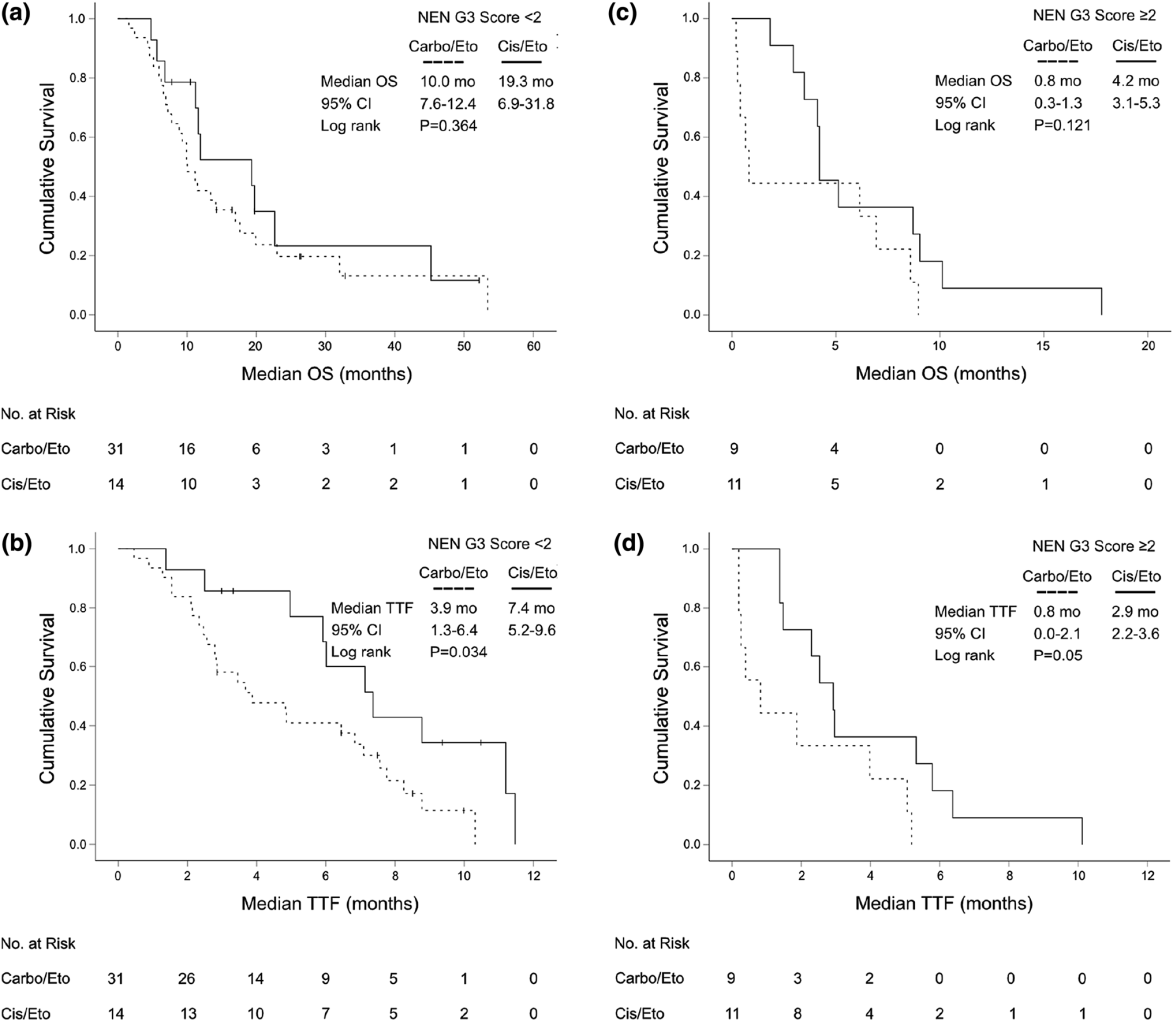
Kaplan–Meier plot for overall survival (OS) and time-to-treatment-failure (TTF) from start of palliative first-line therapy with carboplatin/etoposide vs cisplatin/etoposide in low (**a**, **b**) and poor risk (**c**, **d**) “NEN G3 Score” subgroups (low risk: NEN G3 Score < 2; poor risk: NEN G3 Score ≥ 2)

As the majority of patients had received first-line therapy with platinum/etoposide, we evaluated the NEN G3 Score separately for this subgroup (*N* = 65). Again, a NEN G3 Score < 2 significantly associated with superior median OS (11.6 months, 95% CI 8.8–14.4 vs 4.2 months, 95% CI 4.0–4.4) (HR 4.4, 95% CI 2.4–8.1, *P* < 0.001). The NEN-G3 Score could also predict TTF: Patients with low risk (NEN-G3 Score < 2) had a median TTF of 5.9 months (95% CI 4.0–7.9), whereas patients in the poor risk group (NEN-G3 Score ≥ 2) had only a median TTF of 2.3 months (95% CI 0.9–3.7) (HR 2.7, 95% CI 1.5–4.7, *P* = 0.001) (Suppl. Figure 3). Next, we analyzed if the more aggressive cytotoxic regimen cisplatin/etoposide could revert the negative impact of the high NEN G3 Score in our patient population. For this, we grouped the patients with NEN G3 Score ≥ 2 into two treatment groups (cisplatin vs. carboplatin). However, the more intense chemotherapeutic regimen cisplatin/etoposide could not significantly improve the median OS or the TTF of the poor risk group compared to carboplatin/etoposide. However, median TTF in the low risk group was significantly prolonged with the more intense regimen cisplatin/etoposide (7.4 months, 95% CI 5.2–9.6 vs 3.9 months, 95% CI 1.3–6.4, *P* = 0.034), but did not translate into a significantly prolonged median OS (*P* = 0.364) (Fig. 6).

## Discussion

The prognosis of patients with metastatic GEP-NEN G3 is poor, and no relevant progress has been made over the past decades. Chemotherapy with platinum/etoposide still is recommended as first-line therapy, and no further-line therapies have been formally established in this rare entity (Ilett et al. 2015). GEP-NEN G3 comprise a heterogeneous group concerning prognosis and response to chemotherapy, which potentially introduced confounders and biases in clinical studies of novel systemic therapies. Against this background, we set out to establish a pragmatic prognostic score for stratification in future clinical trials as well as for clinical decision-making with contemporary standard therapies. Next to standard clinical parameters focused on systemic markers of inflammation which is established as a hallmark of cancer (Hanahan and Weinberg 2011).

Our analysis is based on a retrospectively identified patient cohort that was treated at a major academic comprehensive cancer center. The vast majority of patients received first-line treatment with platinum/etoposide, which is consistent with current treatment guidelines. But, in second-line treatment, topotecan (*N* = 20, 37.0%) and the ACO protocol (*N* = 10, 18.5%) were the most commonly applied regimens according to SCLC treatment, which is no longer recommended in the updated NCCN guidelines (Shah et al. 2021). Median OS (9.0 months, 95% CI 7.0–11.1) was comparable to the NORDIC NEC study (11 months) but was lower compared to two other larger retrospective studies (15.6–22.8 months) (Sorbye et al. 2013; Heetfeld et al. 2015; Walter et al. 2017). Notably, these studies included about three times larger patient populations. Despite enrichment of high-risk patients (Ki-67 > 55% in 66.7% of patients) in our real-life cohort, the median TTF of 4.9 months (95% CI 3.4–6.4) with first-line therapy was in the range of PFS results reported for the control arms of recent phase III studies in SCLC (Horn et al. 2018; Paz-Ares et al. 2019), which is generally used as reference entity for the very rare GEP-NEN G3. Our data are also comparable with a retrospective study of Fisher et al. who reported a median PFS of 4.5 months in first-line treatment and a median OS of 12.3 months for patients with G3 NEN (Fisher et al. 2019). A retrospective study by Jann et al. of a heterogeneous cohort of 105 patients with an extra-pulmonary NEN G3 reported a DCR of 75.2% and an ORR of 58.1% to first-line chemotherapy (Jann et al. 2020). In our cohort, no difference was observed between cisplatin- and carboplatin-based first-line therapy (*P* = 0.091); however, by the retrospective nature of our analysis, confounding cannot be ruled out. We confirmed higher response rates in patients with highly proliferative (Ki-67 > 55%) GEP-NEN G3, as described previously by the NORDIC NEC study and the data from Jann et al. (Sorbye et al. 2013; Jann et al. 2020). Consequently, our findings underline the high medical need for innovative approaches aiming to improve outcomes of patients with GEP-NEN G3.

Due to the rarity of the disease, it is expected that future clinical studies will enroll relatively small cohorts, and may even be single-armed trials. Accordingly, a precise definition of patient cohorts is of utmost importance for valid interpretation of study outcomes. While prior analyses have largely relied on clinical parameters, here we have explored the additional contribution of systemic markers of inflammation that can be derived from laboratory analyses routine performed in clinical practice. At univariate analysis, ECOG, Ki-67 index, LDH and all SIR markers (the absolute lymphocyte and neutrophil count, NLR, LMR, PLR and CRP) correlated with OS. Of those, the pretreatment absolute lymphocyte count emerged as important marker at multivariate analysis. Integrating the pretreatment absolute lymphocyte count with ECOG PS and LDH activity, we have developed a pragmatic score which effectively separates patients with superior and inferior OS and TTF from first-line therapy with platinum/etoposide from this heterogeneous and rare cancer entity. This score may serve patient stratification in future prospective clinical trials. It may also guide shared clinical decision-making with contemporary therapies.

## Supplementary Information

Below is the link to the electronic supplementary material.

Supplementary file1 (PDF 419 KB)

## Data Availability

The datasets generated during and/or analyzed during the current study are available from the corresponding author on reasonable request.
