# Supplementary material for: Identification of a new prognostic score for patients with high-grade metastatic GEP-NEN treated with palliative chemotherapy
Source: J Cancer Res Clin Oncol. 2022 Sep 8;149(8):4315–25. doi: 10.1007/s00432-022-04314-5 (PMC10349702; doi:10.1007/s00432-022-04314-5)
Supplement: Supplementary file 1 — Supplementary file1 (PDF 419 KB) [file 432_2022_4314_MOESM1_ESM.pdf]

Suppl. Table 1 Initially measured blood parameters.

Parameter (unit)	Median (range)
LDH (U/l)	298 (149 - 679)
> 1.5 ULN: N=31 (40%)	
Lymphocytes (/nl)	1.4 (0.4 - 9.3)
Neutrophils (/nl)	6.3 (1.3 - 21.1)
Neutrophil-lymphocyte ratio (NLR)	4.1 (0.5 - 23.8)
Lymphocyte-monocyte ratio (LMR)	1.8 (0.4 - 11.6)
Platelet-lymphocyte ratio (PLR)	202 (33.9 - 612.8)
CRP (mg/dl)	1.8 (0.5 - 24.2)

ULN upper limit of normal, *CRP* C-reactive protein.

Suppl. Table 2 Distribution of palliative chemotherapy protocols (N=78).

		%	N
First-line	Etoposide/Platinum	83.3	65
N=78	Etoposide/Cisplatin	32.1	25
	Etoposide/Carboplatin	51.3	40
	Other (FOLFOX, FOLFIRINOX, Carboplatin mono, Cisplatin mono, ACO, CapOx, Topotecan)	16.7	13
Second-line	Topotecan	37.0	20
N=54 (69.2% of first line-patients)	ACO	18.5	10
	FOLFOX/FOLFIRI/FOLFIRINOX	16.7	9
	Etoposide/Platinum	16.7	9
	Other	11.1	6
Third-line	ACO	26.7	8
N=30 (38.5% of first-line patients)	Topotecan	13.3	4
	Capecitabine/Temozolomide	10.0	3
	FOLFOX/FOLFIRI	16.7	5
	Other	33.3	10
≥ Fourth line (N=18, 23.1% of first-line patients)			

FOLFIRINIOX irinotecan, oxaliplatin, fluorouracil and leucovorin, *FOLFOX* oxaliplatin, fluorouracil and leucovorin; *FOLFIRI* irinotecan, fluorouracil and leucovorin, *CapOx* capecitabine/oxaliplatin, *ACO* doxorubicin, cyclophosphamide, vincristine

Suppl. Table 3 Response rates for patients eligible for RECIST 1.1 evaluation for a) sequential palliative chemotherapy (N=52) and b) for first-line treatment with platinum/etoposide according to the Ki-67 index (N=47).

a)	%	N
1st line (N=52)		
CR	3.9	2
PR	30.8	16
SD	42.3	22
PD	23.0	12
ORR 1st	34.6	18
DCR 1st	76.9	40
2nd line (N=21)		
CR / PR	0	0
SD	47.6	10
PD	52.4	11
3rd line (N=11)		
CR /PR	0	0
SD	27.3	3
PD	72.7	8

b)	Ki-67 ≤55% (N=18)		Ki-67 >55% (N=29)		Odds ratio (95% CI)	P-value
	%	N	%	N		
CR	5.6	1	3.5	1		
PR	-	-	44.8	13		
SD	55.6	10	34.5	10		
PD	38.9	7	17.2	5		
ORR	5.6	1	48.3	14	15.9 (1.9-135.4)	**0.003**
DCR	61.1	11	82.8	24	3.1 (0.8-11.8)	0.168

ORR overall response rate, *DCR* disease control rate, *CR* complete response, *PR* partial response, *SD* stable disease, *PD* progressive disease.

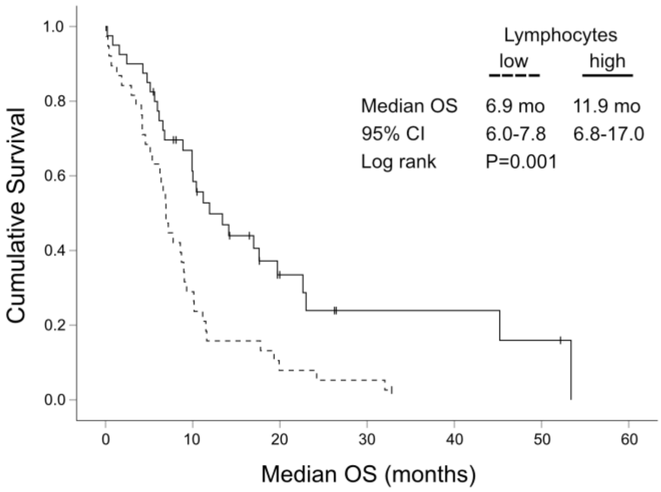

No. at Risk

Lymphocytes high	40	22	7	3	3	2	0
Lymphocytes low	38	11	3	2	0	0	0

Suppl. Fig. 1 Kaplan-Meier plot for median overall survival (OS) for patients with high and low absolute lymphocyte counts in their baseline blood results (N=78).

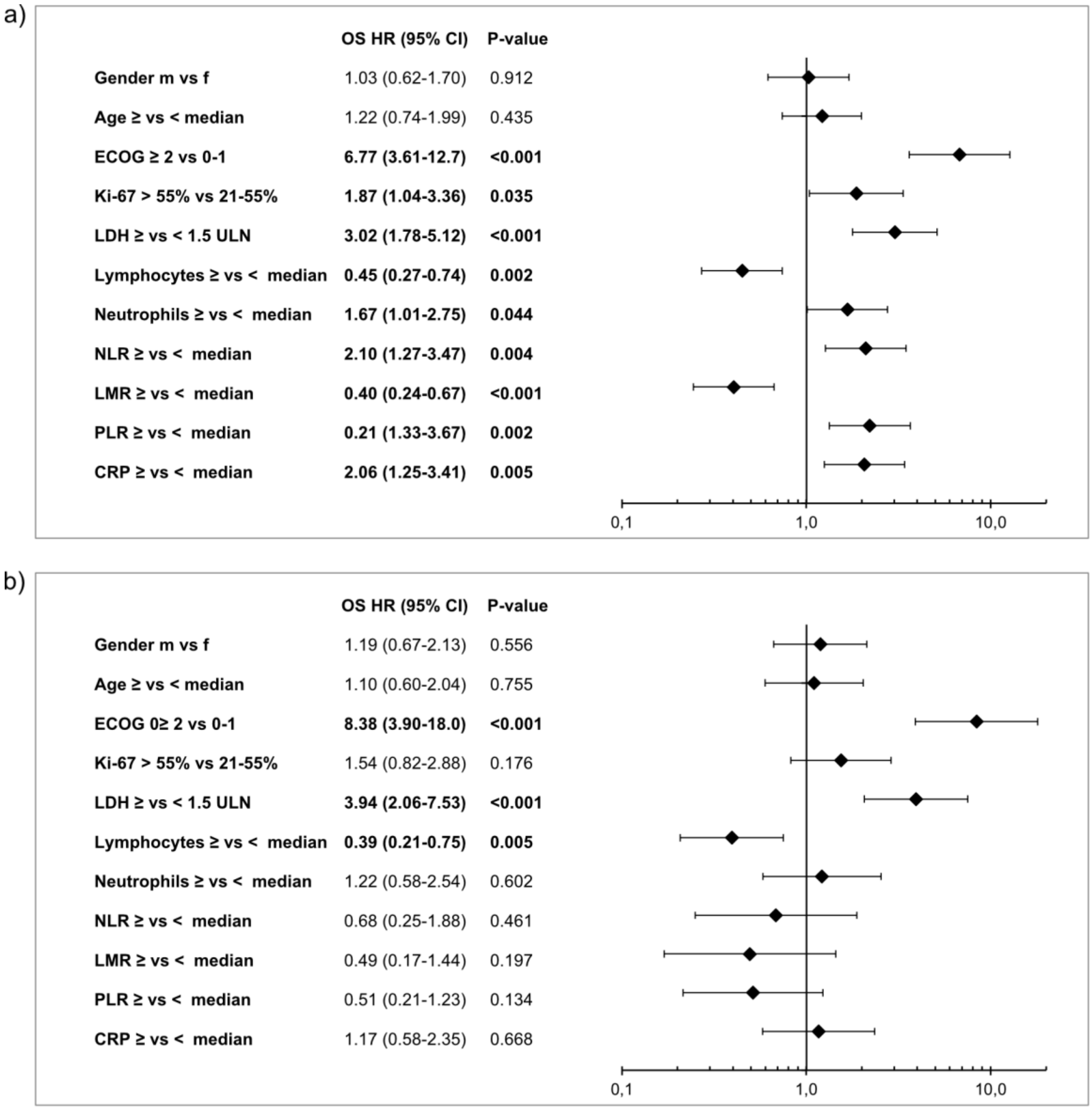

Suppl. Fig. 2 Forrest plot for the a) univariate and b) multivariate analysis of overall survival (OS) from start of palliative treatment. *ECOG* Eastern Cooperative Oncology Group, *ULN* upper limit of normal, *LDH* lactate dehydrogenase, *CRP* C-reactive protein, *HR* hazard ratio, *OS* overall survival, *CI* confidence interval, *m* male, *f* female

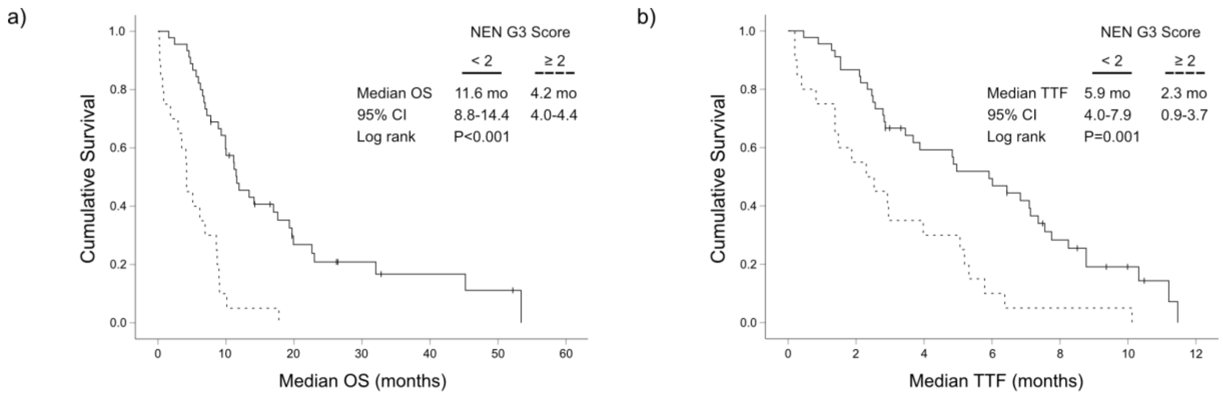

a) No. at Risk

NEN G3 Score <2	45	26	9	5	3	2	0
NEN G3 Score ≥2	20	2	0	0	0	0	0

b) No. at Risk

NEN G3 Score <2	45	39	24	20	10	4	0
NEN G3 Score ≥2	20	11	6	2	1	1	0

Suppl. Fig. 3 Kaplan-Meier plot for a) median overall survival (OS) and b) time-to-treatment failure (TTF) for patients with a high (⩾2 prognostic factors) and low (<2 prognostic factors) “NEN G3 Score” which received platinum/etoposide as first-line palliative treatment (N=65).
